# Does the order matter? Comparing the order of stem placement and fracture reduction in revision total hip arthroplasty for Vancouver B2 and B3 periprosthetic femur fractures

**DOI:** 10.1007/s00402-026-06351-y

**Published:** 2026-06-03

**Authors:** Sophia S. Antonioli, Farouk Khury, Mitchell F. Kennedy, Muhammad A. Haider, Alexander Duke, Ran Schwarzkopf, Vinay K. Aggarwal

**Affiliations:** 1 Department of Orthopedic Surgery, New York University Langone Orthopedic Hospital, New York, USA; 2 The Ruth and Bruce Rappaport Faculty of Medicine, Technion—Israel Institute of Technology, Haifa, Israel; 3 Division of Orthopedic Surgery, Rambam Health Care Campus, Haifa, Israel

**Keywords:** Periprosthetic femur fracture, Revision total hip arthroplasty, Subsidence, Vancouver classification

## Abstract

**Background:**

Vancouver B2 and B3 periprosthetic femur fractures (PPFF) have posed significant treatment challenges due to stem instability and lack of adequate femoral bone stock. This study investigated subsidence, survivorship, and outcomes of Vancouver B2 and B3 fractures, based on the order in which revision stem placement and fracture reduction occurred during revision total hip arthroplasty (rTHA).

**Methods:**

This retrospective, cohort study included 46 rTHAs between June 2011 and April 2023. Included patients underwent rTHA for Vancouver B2 or B3 PPFF with minimum one-year radiographic and two-year clinical follow-up. All patients were treated with diaphyseal-engaging tapered fluted titanium stems and stem modularity decisions were based on surgeon preference. Cohorts were separated based on if stem placement (SF, *n* = 19), or fracture reduction (RF, *n* = 27) occurred first. Patient demographics, intraoperative information, and clinical and radiographic outcomes were collected.

**Results:**

The SF and RF cohort showed no statistically significant differences in rate of subsidence ≥5 mm [26.3%[SF], 22.2%[RF], *P* = 0.749), rate of subsidence ≥ 10 mm (15.8%[SF], 14.8%[RF], *P =* 0.928), nor average subsidence (4.1 mm[SF], 4.4 mm[RF], *P* = 0.861). We found no statistically significant differences in surgery-related clinical outcomes or all-cause revision rates within a two-year follow-up period. The groups demonstrated comparable rates of procedure-related 90-day emergency department visits(*P* = 0.370) and readmissions(*P* = 0.712). The SF group underwent four revisions for three PJIs and one acetabular component aseptic loosening. The RF cohort underwent four revisions for one acetabular component aseptic loosening, one dislocation, one PPFF, and one PJI. Rates of all-cause revision were comparable(*P =* 0.583). There was one case within the RF cohort to explant the trochanteric plate with no revision of arthroplasty components.

**Conclusions:**

The present analysis suggests the order in which intraoperative femoral stem implantation and fracture reduction occurs does not affect short-term clinical and radiographic outcomes. This intraoperative decision should be based upon patient anatomy, fracture patterns, and surgeon discretion.

## Introduction

With the projected surge in patients undergoing total hip arthroplasty (THA), set to increase by a staggering 174% by 2030 [1, 2], there arises a resultant demand for revision THA (rTHA). Much of the increased demand for rTHA stems from an increasing proportion of patients sustaining periprosthetic femur fracture (PPFF) [3]. Although various implant designs and surgical techniques have been proposed for rTHAs due to PPFF [4–8], many questions remain including whether the order in which surgeons should implant the femoral stem versus reduce the femoral fracture influences ultimate outcomes.

The Vancouver classification for PPFF has been extrapolated to modern-day cementless implants and helps guide treatment of grossly loose femoral stems, which are now most often revised with the use of a titanium diaphyseal engaging tapered fluted stem [4, 9, 10]. Although there is a high treatment success rate when using these stems in rTHA, there remains a risk of failure and progression to challenging re-revision operations. Although there has been much debate regarding the use of modular versus monoblock tapered fluted stems in rTHA [11–15], the order in which the stem is placed versus the fracture is reduced in PPFF has not been investigated in the current literature. Because the success of these operations is based on both stem fixation and fracture union, this order may ultimately impact how these cases fail, including stem subsidence, failure of osseointegration, limb shortening with resultant pain from altered hip biomechanics, and hip instability [16].

With these factors in mind, we sought to investigate the impact of the order of revision stem placement and fracture reduction on outcomes of Vancouver B2 and B3 periprosthetic femur fractures. Specifically, we compared the two cohorts with regards to radiographic subsidence, implant survivorship at 2 years, and clinical outcomes such as complications and re-revision THA.

## Methods

### Study design and cohort selection

This was an institutional review board approved research protocol (protocol #i17-01223_CR5). A retrospective review was conducted on 2,226 patients who underwent rTHA between June 2011 and April 2023 at two urban, high-volume, academic hospital. Patients were included in the study if they underwent rTHA for a primary diagnosis of Vancouver B2 or B3 PPFF [17] and had a minimum of two-years of clinical follow-up and one-year radiographic follow-up. The primary indication for rTHA as periprosthetic fracture was captured using International Classification of Diseases 10 (ICD-10) code (M97) and manual chart review. Of the starting cohort of 2,226 patients who underwent rTHA, 2,050 patients were excluded due to having an indication for revision other than PPF, 98 were excluded due to inadequate clinical or radiographic follow-up, and 78 were excluded due to the reported fracture being acetabular or not classified as Vancouver B2 or B3 PPFF. The final study cohort consisted of 46 patients who were then divided into two cohorts — one cohort that underwent femoral stem placement prior to PPFF reduction (stem first, SF) (*n* = 19) and one cohort that underwent PPFF reduction prior to femoral stem placement (reduced first, RF) (*n* = 27).

### Data collection and outcome measures

Our institution’s Electronic Medical Record (EMR) (Epic Caboodle. Version 15; Verona, Wisconsin, USA) was utilized to collect patient demographics including age, sex, race, BMI, smoking status, insurance coverage, and American Society of Anesthesiologists (ASA) score. Sex, as described throughout the study, refers to the patient’s sex assigned at birth. Perioperative data including surgical time, length of stay (LOS), and discharge disposition were also collected. Surgical time was defined as the number of minutes from initial skin incision to skin closure as documented in the EMR. LOS was defined as the number of days spent in the hospital following surgery. Information regarding fixation method of the primary THA was obtained through review of preoperative radiographs and available clinical documentation. Preoperative radiographs were retrospectively reviewed to further characterize fracture morphology. In addition to Vancouver classification, fractures were qualitatively categorized by pattern (oblique, spiral, comminuted, or transverse), consistent with prior studies utilizing descriptive morphology as an adjunct to established classification systems [18]. Chart review of operative notes and radiographs was conducted to collect implant data including order of intraoperative stem placement and fracture reduction, the use of additional fixation hardware during the rTHA, and femoral stem design (modular versus monoblock) (Table 3).

**Fig. 1:**
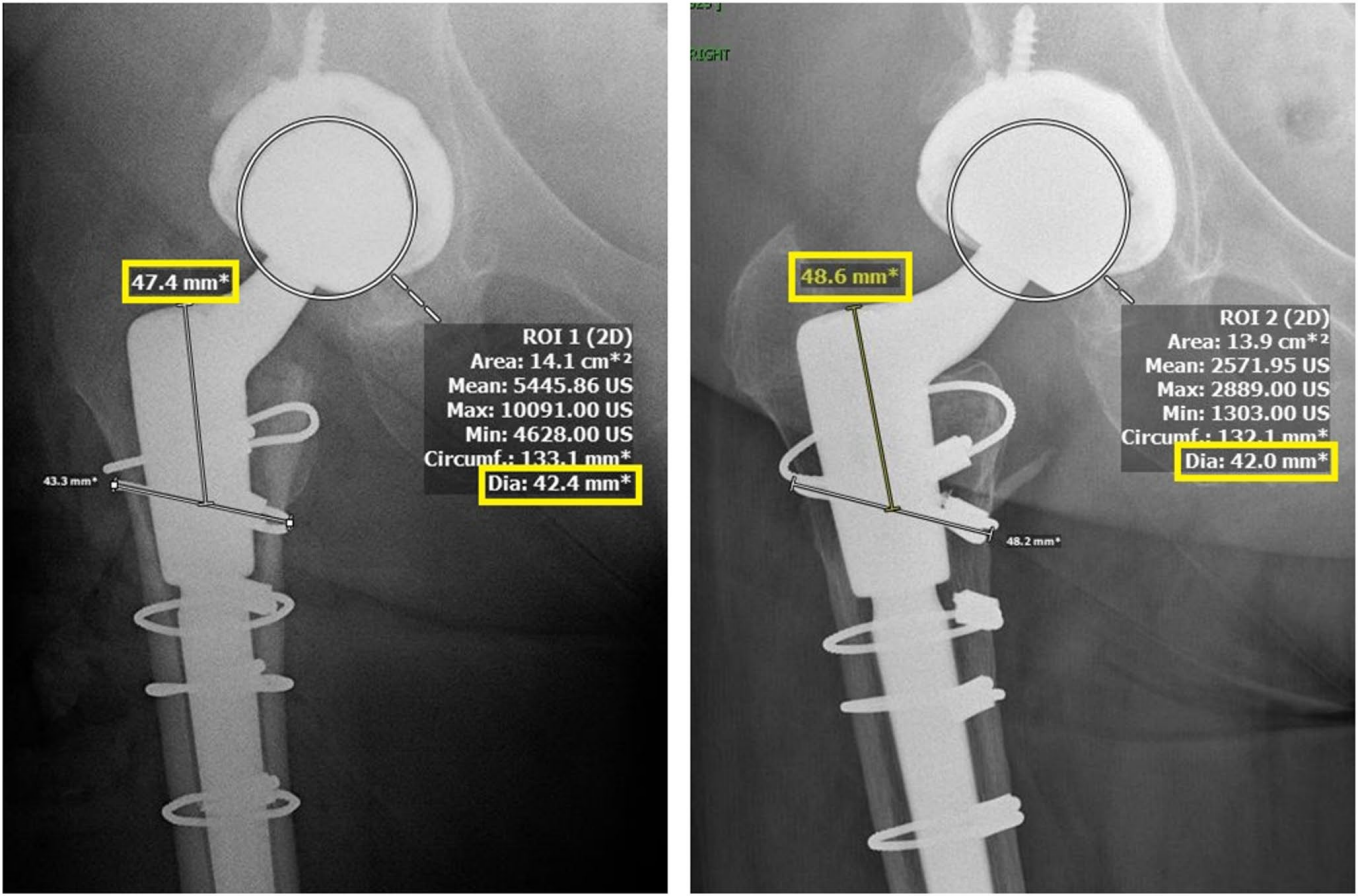
Example Subsidence Measurement. Intraoperative AP radiograph (left), *≥* 1-year postoperative radiograph (right)

AP radiographs were reviewed to measure the distance in millimeters (mm) between a fixed landmark on the prosthesis (the shoulder of the femoral stem) and a fixed landmark on the femur (most proximal cerclage cables if present, center of lesser trochanter if cables were not present) [13, 16, 19–21]. The difference in this measurement on immediate postoperative x-rays and at least one-year postoperative x-rays, adjusted for magnification, was considered the subsidence for each patient. Subsidence was measured by two authors (SA and FK, intraclass correlation coefficient of 97%, Fig. 1). The following equation was used to establish subsidence values:

$$ \begin{gathered} {\text{Subsidence = [(Head diameter A / Head diameter B) }} \hfill \\ \quad \quad \quad \quad \quad \quad \times {\text{ (Subsidence B)]}} \hfill \\ \quad \quad \quad \quad \quad \quad {\text{ = [(42}}{\text{.0 / 42}}{\text{.4) x 48}}{\text{.6] }} \hfill \\ \end{gathered} $$

Lastly, clinical outcomes were collected from the institutional EMR including 90-day emergency department (ED) visits and 90-day readmissions. Revision surgeries were also collected and confirmed by manual chart review. A revision was defined as a return to the operating room during which hardware was replaced or removed.

### Data analyses

The primary outcomes of this study were the differences in rate of subsidence and mean amount of subsidence between the two cohorts – one that underwent femoral stem placement prior to PPFF fracture reduction (SF), and another that underwent PPFF reduction prior to femoral stem placement (RF). All patient data was stored in an encrypted Excel file (Microsoft, Redmond, Washington, USA), and saved in a secure data drive using Microsoft SQL Server Management Studio 2017 (Microsoft, Redmond, Washington, USA). Baseline characteristics of patients were represented as frequencies with percentages for categorical variables and means with standard deviations for continuous variables. Statistical differences in categorical variables and continuous variables were detected using *chi*-squared (x^2^) tests and independent sample *t*-tests respectively. Significance was set as a *P*-value less than 0.05. All statistical analyses were conducted using SPSS Statistics (Version 28; IBM Corporation, Armonk, New York, USA).

## Results

### Baseline characteristics

The final study population consisted of 46 revision THAs, 19 who underwent femoral stem placement prior to PPFF reduction (SF) and 27 who underwent PPFF reduction prior to femoral stem placement (RF). There were no statistically significant differences in patient demographics including mean age, sex, mean BMI, race, smoking status, and insurance type between the two cohorts. The cohorts also had similar distributions of ASA status and laterality of procedure. Each cohort consisted of one patient who had undergone a previous rTHA before the rTHA due to PPFF currently in question. Prior primary THA fixation was exclusively uncemented in all 19 SF patients (100%), whereas the RF cohort consisted of 23 uncemented (85.2%) and 4 cemented (14.8%) primary stems. There was no significant difference in fracture pattern distribution between the SF and RF cohorts (*P* = 0.435), with both groups demonstrating a predominance of spiral fractures and similar proportions of comminuted patterns. A detailed account of patient demographics is reported in Table 1.

**Table 1:** Patient Baseline Characteristics

Mean age, years ± SD	Stem placed first *n* = 19	Fracture reduced first *n* = 27	*P*-Value
	72.7 ± 8.7	70.1 ± 9.7	0.177
Sex, n (%)			0.749
Male	5 (26.3)	6 (22.2)	
Female	14 (73.7)	21 (77.8)	
Mean BMI, km/m^2^ ± SD	28.2 ± 6.2	25.9 ± 6.6	0.120
Race, n (%)			0.108
White	17 (89.5)	24 (88.9)	
Black or African American	2 (10.5)	1 (3.7)	
Other	0 (0.0)	2 (7.4)	
Smoking Status, n (%)			0.759
Never	9 (47.4)	13 (48.1)	
Former	9 (47.4)	11 (40.8)	
Current	1 (5.2)	3 (11.1)	
Insurance, n (%)			0.182
Medicare	16 (84.2)	18 (66.7)	
Commercial	3 (15.8)	9 (33.3)	
ASA Score, n (%)			0.841
I	0 (0.0)	1 (3.7)	
II	12 (63.2)	15 (55.6)	
III	6 (31.6)	9 (33.3)	
IV	1 (5.2)	2 (7.4)	
Laterality, n (%)			0.526
Right	13 (68.4)	16 (59.3)	
Left	6 (31.6)	11 (40.7)	
Previously Underwent rTHA, n (%)	1 (5.3)	1 (3.7)	0.798
Fracture Pattern, n (%)			0.435
Oblique	0 (0.0)	2 (7.4)	
Spiral	14 (73.7)	16 (59.3)	
Comminuted	5 (26.3)	9 (33.3)	

*SD* standard deviation, *BMI* body mass index, *ASA Score* American society of anesthesiologists score

### Perioperative and implant data

There were no significant differences in perioperative outcomes between the two cohorts. The SF and RF groups had similar mean surgical times (*P =* 0.149) and discharge dispositions (*P =* 0.838). The SF cohort was discharged sooner after surgery than the RF cohort, although this finding did not reach statistical significance (4.8 days [SF], 6.6 days [RF], *P* = 0.093).

**Fig. 2:**
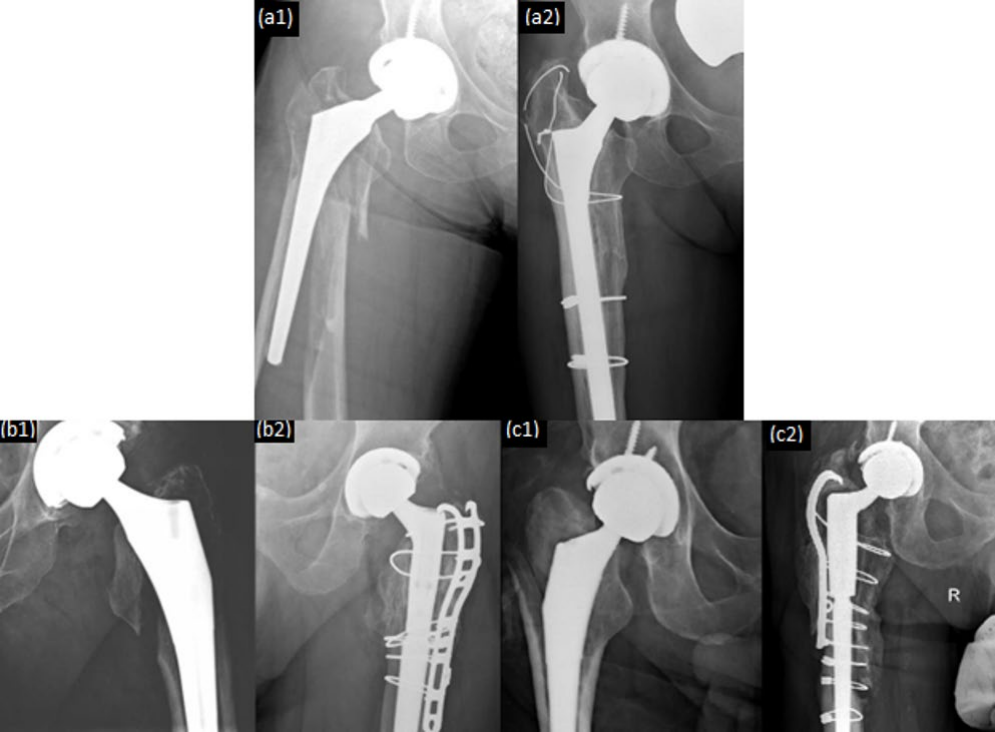
Preoperative and Postoperative Anteroposterior Radiographs. (a1) Fracture fixed first, monoblock (pre-revision). (a2) Fracture fixed first, monoblock (post-revision). (b1) Stem placed first, modular (pre-revision). (b2) Stem placed first, modular (post-revision). (c1) Fracture fixed first, modular (pre-revision). (c2) Fracture fixed first, modular (post-revision)

All 46 patients included in the study were treated with diaphyseal engaging tapered fluted titanium stems and the decision regarding stem modularity was based on surgeon discretion. The difference in stem modularity between the two cohorts reached statistical significance (*P* = 0.036). Monoblock stems were utilized more frequently in the stem-first cohort (68.4% vs. 37.0%), while modular stems were more common in the fracture-reduced-first cohort (31.6% vs. 63.0%) (Fig. 2).

There was no difference in frequency in which the cohorts required additional hardware such as cables and trochanteric plates (*P* = 0.186). Three patients (15.8%) of the SF cohort did not require cables nor trochanteric plates, whereas all patients in the RF cohort require the use of one or both. Within the SF group 68.4% of patients utilized cables with no trochanteric plate versus 74.1% of the RF group. Of the SF patients, 15.8% utilized a trochanteric plate and cables versus 25.9% of the RF patients (Tables 2 and 3).

**Table 2:** Perioperative Data

Mean surgical time, minutes [range]	Stem placed first *n* = 19	Fracture reduced first *n* = 27	*P*-Value
	138.9 [75–207]	159.9 [69–464]	0.149
Mean Length of Stay, days [range]	4.8 [1–18]	6.6 [1–20]	0.093
Discharge Disposition, n (%)			0.838
Home	7 (36.9)	8 (29.6)	
SNF	10 (52.6)	15 (55.6)	
ARF	2 (10.5)	4 (14.8)	
SNF, Skilled Nursing Facility; ARF, Acute Rehabilitation Facility			

**Table 3:** Implant Data

Additional hardware, *n* (%)	Stem placed first *n* = 19	Fracture reduced first *n* = 27	*P*-Value
			0.186
None	3 (15.8)	0 (0.0)	
Cables	13 (68.4)	20 (74.1)	
Trochanteric Plate + Cables	3 (15.8)	7 (25.9)	
Stem Design, n (%)			0.036
Monoblock	13 (68.4)	10 (37.0)	
Modular	6 (31.6)	17 (63.0)	

*SD* standard deviation, *PPFF* periprosthetic femur fracture, *mm* millimeters

### Subsidence

Regarding subsidence, the primary outcome of the study, there were no significant differences in rate of occurrence of subsidence more than or equal to 5 mm between the two cohorts with 26.3% of cases within the SF cohort and 22.2% of cases in the RF cohort exhibiting radiographically identified subsidence ≥ 5 mm at more than one-year post-operatively (*P =* 0.749). Additionally, there was no significant difference in the mean subsidence between the cohorts when only averaging cases that did exhibit subsidence ≥ 5 mm (10.27 mm [SF], 13.55 mm [RF], *P =* 0.459). When evaluating cases of subsidence more than or equal to 10 mm, we found 15.8% of cases within the SF cohort and 14.8% of cases in the RF cohort met this criterion for radiographically identified subsidence more than one-year post-operatively (*P =* 0.928). When averaging the subsidence cases greater than 10 mm for each cohort, we found no significant difference (13.44 mm [SF], 17.72 mm [RF], *P =* 0.364). The average subsidence of all SF cases and RF cases were 4.05 mm and 4.35 mm, respectively (*P =* 0.861). The average radiographic follow-up was 1.93 years for the SF cohort and 1.62 years for the RF cohort (*P =* 0.243). Full reporting of femoral stem subsidence data can be found in Table 4.

**Table 4:** Femoral Stem Subsidence

Mean Subsidence, mm ± SD	Stem placed first *n* = 19	Fracture reduced first *n* = 27	*P*-Value
	4.05 ± 4.54	4.35 ± 6.33	0.861
Subsidence ≥ 5 mm, n (%)	5 (26.3)	6 (22.2)	0.749
Subsidence ≥ 10 mm, n (%)	3 (15.8)	4 (14.8)	0.928
Mean Time to Radiographic Follow-Up, years ± SD	1.93 [1.03–3.86]	1.62 [0.99–3.24]	0.243

* Mm* millimeters, *SD* standard deviation

As previously stated, there was an uneven distribution of modular versus monoblock stem usage between the SF and RF cohorts. To further understand the influence of this variable, we conducted a subanalysis to stratify each cohort by the type of stem received. Within the SF cohort, six patients received modular stems and 13 patients received monoblock stems. When comparing the patients within the SF cohort who received either stem, there were no significant differences regarding the frequency of subsidence ≥ 5 mm (*P* = 0.637), frequency of subsidence ≥ 10 mm (*P* = 0.154), nor overall average subsidence (*P* = 0.318). Within the RF cohort, 17 patients received modular stems and 10 patients received monoblock stems. Our analysis also revealed no significant difference within the RF cohort regarding stem design at the various metrics: frequency of subsidence ≥ 5 mm (*P* = 0.088), average subsidence ≥ 5 mm (*P* = 0.697), frequency of subsidence ≥ 10 mm (*P* = 0.088) average subsidence ≥ 10 mm (*P* = 0.982), nor overall average subsidence (*P* = 0.119). A full evaluation of subsidence stratified by the type of stem implanted can be found in Table 5.

**Table 5:** Femoral Stem Subsidence Stratified by Stem Type

	Stem placed first *n* = 19			Fracture reduced first *n* = 27		
	Modular *n* = 6	Monoblock *n* = 13	*P*-Value	Modular *n* = 17	Monoblock *n* = 10	*P*-Value
Subsidence ≥ 5 mm						
Present, n (%)	2 (33.3)	3 (23.1)	0.637	2 (11.8)	4 (40.0)	0.088
Mean, mm ± SD	13.49 ± 3.47	8.12 ± 4.53	0.262	11.33 ± 8.78	14.66 ± 9.33	0.697
Subsidence ≥ 10 mm						
Present, n (%)	2 (33.3)	1 (7.7)	0.154	1 (5.9)	3 (30.0)	0.088
Mean, mm ± SD	13.49 ± 3.71	13.34 ± NA	0.979	17.54 ± NA	17.78 ± 8.49	0.982
Mean Subsidence, mm ± SD	5.63 ± 6.41	3.33 ± 3.47	0.318	2.89 ± 4.11	6.84 ± 8.66	0.119

*Mm* millimeters, *SD* standard deviation

### Clinical outcomes

There was a significant difference in rate of all-cause 90-day ED visits between the groups (15.8% [SF], 0% [RF], *P =* 0.033). All three cases of 90-day ED visits occurred within the stem placed first group. These three patients reported to the ED for the concerns of post-operative hip pain, anorexia, and weakness. When the visit complaints were subcategorized as related or unrelated to the procedure, the previously observed statistical difference did not persist (related to the procedure *P =* 0.370; unrelated to the procedure *P =* 0.181). There was no significant difference in rate of all-cause 90-day readmissions (21.1% [SF], 11.1% [RF], *P =* 0.355), nor a difference when the reason for readmission was stratified by related (*P =* 0.712) or unrelated (*P =* 0.356) to the procedure. Regarding readmissions related to the procedure, two patients within the SF cohort were readmitted due to PJI, one patient within the RF cohort was readmitted due to PJI, and one patient within the RF cohort was readmitted for a second PPFF.

There was no significant difference between the rate of all-cause revision between the groups (21.1% [SF], 14.8% [RF], *P =* 0.583). The four cases of revision within the stem placed first cohort were due to one case of aseptic loosening of the acetabular component that occurred 1.3 years after the index revision for PPFF and three cases of PJI. The four cases of revision within the RF cohort were due to one case of aseptic loosening of the acetabular component with symptomatic leg length discrepancy, one case of instability/recurrent dislocation, one case of recurrent PPFF, and one case of PJI. Within this cohort the revision for acetabular component loosening and leg length discrepancy occurred 4.2 years after the index revision for PPFF, the revision for instability occurred 1.1 years after, and the case of recurrent PPFF occurred 0.4 years after. Although three patients (15.8%) within the SF cohort versus only one patient (3.7%) within the RF cohort experienced PJI, this difference was not statistically significant (*P =* 0.152). In addition to the revision procedures in which implants were exchanged, there was one reoperation within the RF cohort. This patient continued to experience significant hip pain and ultimately underwent explant of the trochanteric plate without exchange of arthroplasty implants 3.1 years after the index revision for PPFF. Full reporting of clinical outcomes can be found in Table 6.

**Table 6:** Clinical Outcomes

90-Day ED visits, *n* (%)	Stem placed first *n* = 19	Fracture reduced first *n* = 27	*P*-Value
	3 (15.8)	0 (0.0)	0.033
Related to Procedure	1 (5.3)	0 (0.0)	0.370
Hip pain	1 (5.3)	0 (0.0)	
Unrelated to Procedure	2 (10.5)	0 (0.0)	0.181
90-Day Readmissions, n (%)	4 (21.1)	3 (11.1)	0.355
Related to Procedure	2 (10.5)	2 (7.4)	0.712
PJI	2 (10.5)	1 (3.7)	
PPFF	0 (0.0)	1 (3.7)	
Unrelated to Procedure	2 (10.5)	1 (3.7)	0.356
Non-Revision Reoperations, n (%)	0 (0.0)	1 (3.7)	0.999
Trochanteric Plate Explant	0 (0.0)	1 (3.7)	
All-Cause Revisions, n (%)	4 (21.1)	4 (14.8)	0.583
Aseptic Loosening Acetabular Cup	1 (5.3)	1 (3.7)*	
Instability/Recurrent Dislocation	0 (0.0)	1 (3.7)	
PPFF	0 (0.0)	1 (3.7)	
PJI (Two Stage)	3 (15.8)	1 (3.7)	
Time to Clinical Follow-Up (years), mean [range]	3.18 [1.94–7.54]	3.48 [1.98–6.33]	0.292

* ED* emergency department, *PPFF* periprosthetic femur fracture, *PJI* periprosthetic joint infection

*Reported leg length discrepancy at time of revision for acetabular loosening

## Discussion

The surgical management of PPPF can be challenging, particularly in Vancouver B2 and B3 fractures in which the femoral prosthesis is unstable and there may be decreased availability of adequate bone stock to utilize for revision-implant fixation [5, 22]. We therefore set out to investigate the surgical dilemma of whether to implant the femoral stem or reduce the fracture first. This retrospective analysis of 46 rTHAs demonstrated no significant differences in outcomes based on whether femoral stem placement or fracture reduction occurred first intraoperatively.

Since the coining of the Vancouver classification system the strategies for addressing PPPF have largely remained unchanged. Patients with type B2 and B3 fractures are typically treated with revision to a longer modular or monoblock femoral stem bypassing the fracture by at least two cortical diameters [4, 23–25]. Depending on the surgeon’s preference, the fracture is reduced and fixated using various techniques and implants. The decision of whether to implant the femoral stem or reduce the fracture first might be patient-, surgeon- and fracture-specific. Theoretically, more complex fracture patterns may favor initial reduction to facilitate alignment, while cases with reliable distal fixation may permit stem implantation prior to fracture reduction. However, we observed that fracture morphology was similarly distributed between cohorts, and operative reports did not indicate that fracture pattern significantly influenced the intraoperative sequence of reduction and stem placement. These findings suggest that fracture pattern was not a primary determinant of surgical strategy in our cohort and that the decision to reduce first or revise the stem is driven primarily by intraoperative feedback and individual surgeon preference.

There was no statistically significant difference (*P =* 0.186) between the two cohorts when considering the use of additional hardware to the implants. While all the cases that were reduced first required osteosynthesis, fractures that were treated with stem implantation first did not necessitate fixation in 15.8% of the cases (Table 3). This finding might be attributed to the presence of a simpler fracture pattern or to the fact that preliminary stem implantation can indirectly lead to more simple fixation technique with cables, wires, and/or sutures.

Although modular and monoblock femoral stems have exhibited similar survivorship, clinical outcomes and radiographic results in revision THAs [26–28], they were not equally implanted in both groups. Of the stem placed first cohort 68.4% of cases utilized monoblock stems, whereas only 37.0% of the fracture was reduced first cohort utilized monoblock stems (*P* = 0.036) (Table 3). The choice of using monoblock versus modular stem is largely a matter of surgeon preference and skill. While modular stems have in some cases been reported to offer improved intraoperative control of several parameters such as length, anteversion and offset [29–31], their rate of stem subsidence, intraoperative fractures, and implant failure has been reported to be higher than monoblock stems [13, 20, 32]. Monoblock stems, despite their lack of modularity versatility, offer enhanced usability and reduced cost [11, 14, 33, 34]. The fact that these two implants demonstrated a trend in their specific application may simply implicate surgeon preferences as the reason behind this finding, or it may be that monoblock stems are more suitable to use in cases where the stem can be placed first, and modular stems are more suitable in fracture reduced-first PPPF rTHA.

While investigating the rates of subsidence, it seems that current literature is somewhat inconclusive regarding monoblock versus modular femoral stem. Although publications by Thomas et al. and Koutalos et al. report higher subsidence rate (6.9%, and 12.4%, respectively) in monoblock stems [11, 12], a study by Clair et al. found that modular stems had rather significantly higher rate of subsidence compared to monoblock stems in Vancouver B2 and B3 PPPF (29.2% vs. 11.3%, *P* < 0.001) [13]. Furthermore, even when questioning the cut-off threshold for subsidence, Pomeroy et al. reported in 2022 and 2023 that there was no difference in subsidence between both monoblock and modular tapered fluted titanium stems at both 5 and 10 mm cut-off values [35, 36]. Due to this disagreement in the literature, we performed a subanalysis to investigate subsidence in our two primary cohorts stratified by stem type. As previously mentioned, the results of this subanalysis do not implicate stem type as a confounding factor for the present study.

Subsidence of the femoral stem can have serious consequences, potentially leading to acute or future aseptic failure, leg length discrepancy, chronic pain, and hip instability [16, 20, 37]. Previous literature is inconclusive regarding what amount of subsidence is clinically relevant [30, 38], leading to our investigation of multiple cut-off values in addition to the overall average subsidence within the groups. With the stem placed first and fracture reduced first cohorts, 26.3% and 22.2% of patients exhibited subsidence ≥ 5 mm, respectively, and 15.8% versus 14.8% of patients exhibited subsidence ≥ 10 mm, respectively (Table 4). Regardless of the method of evaluation, we found no significant difference in subsidence between the SF and RF cohorts. We there conclude there are no differences in risk of subsidence depending on the order of stem placement and fracture reduction.

Regarding clinical outcomes, our analysis did not reveal any significant differences in the frequency of surgery-related 90-day ED visits or readmissions. Additionally, we found no differences in the rate of all-cause revision. We did, interestingly, find the stem placed first group experienced revision due to PJI more frequently (15.8% versus 3.7%), although this finding was not statistically significant. Another interesting finding is that within the total cohort of 46 patients, two patients required revision for acetabular component loosening and not a single patient required revision for aseptic stem loosening. While investigating 51 re-revision procedures, Yu et al. reported findings consistent with our own, that aseptic acetabular component loosening, PJI, and instability were leading causes for re-revision THA [39]. When considering our entire cohort of patients our findings are also consistent with those of Sarpong et al. who reported an 18% re-revision rate at an average of 2.5 years follow-up [40]. The present study found a re-revision rate of 17.4% with an average of 3.3 years follow-up. While several parameters have been previously investigated as risk factors for a re-revision [41], our study did not analyze these correlations. We do, however, feel confident in reporting no difference in revision rates or other clinical outcomes associated with the order in which the femoral stem is placed, or the fracture is reduced.

### Limitations

This study has several limitations inherent to its retrospective design. First, our assessment of intraoperative details, such as the exact degree of residual stem fixation to isolated bone fragments prior to extraction, was limited to the specific details described in the operative notes. Furthermore, while we qualitatively categorized the gross fracture morphologies, the lack of a standardized classification system for periprosthetic fracture patterns remains a potentially confounding factor. The specific anatomical complexity and pattern of each fracture may have influenced the selected surgical sequence, stem modularity, and use of additional hardware. Future research on this topic should investigate whether PPFF pattern is associated with varying clinical outcomes investigated in the current study. Additionally, when considering subsidence measurements on radiographs, despite an ICC of 97% and additional calculations to account for radiographic magnification, inaccuracies in measurements could exist. Lastly, the relatively small sample size in this study may have limited the ability to find statistically significant results.

## Conclusion

Our retrospective cohort study investigating the impact of the order in which femoral stem placement or fracture reduction occurs in rTHA for Vancouver B2 and B3 PPFF did not find statistically significant differences in occurrence of subsidence ≥ 5 mm, subsidence ≥ 10 mm, or average subsidence measured on radiographs more than one year post-operatively. We found no statistically significant differences in surgery-related clinical outcomes or all-cause revision rates within a two-year follow-up period. Our findings argue that the order in which intraoperative femoral stem implantation and fracture reduction occurs does not affect short-term clinical and radiographic outcomes. We suggest this intraoperative decision should be based upon patient anatomy, fracture patterns, and surgeon discretion.

## Data Availability

No datasets were generated or analysed during the current study.

## References

[1] Shichman I, Roof M, Askew N, Nherera L, Rozell JC, Seyler TM et al (2023) Projections and Epidemiology of Primary Hip and Knee Arthroplasty in Medicare Patients to 2040–2060. JB JS Open Access 8:e2200112. 10.2106/JBJS.OA.22.00112 10.2106/JBJS.OA.22.00112 PMC9974080 36864906

[2] Shichman I, Askew N, Habibi A, Nherera L, Macaulay W, Seyler T et al (2023) Projections and Epidemiology of Revision Hip and Knee Arthroplasty in the United States to 2040–2060. Arthroplast Today 21:101152. 10.1016/j.artd.2023.101152 37293373 10.1016/j.artd.2023.101152 PMC10244911

[3] Schwartz AM, Farley KX, Guild GN, Bradbury TL (2020) Projections and Epidemiology of Revision Hip and Knee Arthroplasty in the United States to 2030. J Arthroplasty 35:S79–85. 10.1016/j.arth.2020.02.030 32151524 10.1016/j.arth.2020.02.030 PMC7239745

[4] Masri BA, Meek RMD, Duncan CP (2004) Periprosthetic Fractures Evaluation and Treatment: Clinical Orthopaedics and. Relat Res 420:80–95. 10.1097/00003086-200403000-00012 10.1097/00003086-200403000-00012 15057082

[5] Rodriguez JA, Berliner ZP, Williams CA, Robinson J, Hepinstall MS, Cooper HJ (2017) Management of Vancouver Type-B2 and B3 Periprosthetic Femoral Fractures: Restoring Femoral Length via Preoperative Planning and Surgical Execution Using a Cementless, Tapered, Fluted Stem. JBJS Essent Surg Techniques 7:e27. 10.2106/JBJS.ST.17.00007 10.2106/JBJS.ST.17.00007 PMC6132713 30233962

[6] Scolaro JA, Schwarzkopf R (2017) Management of Interprosthetic Femur Fractures. J Am Acad Orthop Surg 25:e63–e69. 10.5435/JAAOS-D-15-00664 28252475 10.5435/JAAOS-D-15-00664

[7] Rozell JC, Delagrammaticas DE, Schwarzkopf R Interprosthetic femoral fractures: management challenges. ORR 2019;Volume 11:119–128. 10.2147/ORR.S209647 10.2147/ORR.S209647 PMC6754334 31572021

[8] Rozell JC, Donegan DJ (2019) Periprosthetic Femur Fractures Around a Loose Femoral Stem. J Orthop Trauma 33:S10–S13. 10.1097/BOT.0000000000001568 31404038 10.1097/BOT.0000000000001568

[9] Zitsch BP, Byrd JJ, Buckner B, Konigsberg BS, Hartman CW, Tapered (2025) Fluted, Titanium Stems in Revision Total Hip Arthroplasty. Orthopedics 48:79–86. 10.3928/01477447-20250217-01 40052900 10.3928/01477447-20250217-01

[10] Baldwin TJ, Deckard ER, Buller LT, Meneghini RM (2024) Incidence and Predictors of Subsidence Using Modular, Tapered, Fluted Titanium Femoral Stems in Aseptic Revision Total Hip Arthroplasty. J Arthroplasty 39:1304–1311. 10.1016/j.arth.2023.10.057 37924992 10.1016/j.arth.2023.10.057

[11] Thomas J, Shichman I, Ohanisian L, Stoops TK, Lawrence KW, Ashkenazi I et al (2023) Monoblock tapered stems in management of UCS B2 and B3 periprosthetic fractures in revision total hip arthroplasty. Bone Jt Open 4:551–558. 10.1302/2633-1462.48.BJO-2022-0160.R1 37524356 10.1302/2633-1462.48.BJO-2022-0160.R1 PMC10390262

[12] Koutalos AA, Varitimidis S, Malizos KN, Karachalios T (2022) Clinical, functional and radiographic outcomes after revision total hip arthroplasty with tapered fluted modular or non-modular stems: a systematic review. HIP Int 32:475–487. 10.1177/11207000211004383 33829900 10.1177/11207000211004383

[13] Clair AJ, Gabor JA, Patel KS, Friedlander S, Deshmukh AJ, Schwarzkopf R (2020) Subsidence Following Revision Total Hip Arthroplasty Using Modular and Monolithic Components. J Arthroplast 35:S299–303. 10.1016/j.arth.2020.03.008 10.1016/j.arth.2020.03.008 32253066

[14] Nadeau RP, Garbuz DS (2016) Monoblock or modular tapered stems: Making the right choice. Semin Arthroplast 27:261–263. 10.1053/j.sart.2017.03.010

[15] Clair AJ, Cizmic Z, Vigdorchik JM, Poultsides LA, Schwarzkopf R, Rathod PA et al (2019) Nonmodular Stems Are a Viable Alternative to Modular Stems in Revision Total Hip Arthroplasty. J Arthroplasty 34:S292–S296. 10.1016/j.arth.2019.03.007 31010773 10.1016/j.arth.2019.03.007

[16] Parry JA, Hernandez NM, Berry DJ, Abdel MP, Yuan BJ (2018) Risk Factors for Subsidence of Modular Fluted Tapered Stems Used During Revision Total Hip Arthroplasty for Periprosthetic Hip Fractures. J Arthroplast 33:2967–2970. 10.1016/j.arth.2018.05.006 10.1016/j.arth.2018.05.006 29859724

[17] Rayan F, Dodd M, Haddad FS (2008) European validation of the Vancouver classification of periprosthetic proximal femoral fractures. J Bone Joint Surg Br Volume 90–B:1576–1579. 10.1302/0301-620X.90B12.20681 10.1302/0301-620X.90B12.20681 19043127

[18] SATTAR A, KÄRRHOLM J, MÖLLER M (2023) Fracture pattern and risk factors for reoperation after treatment of 156 periprosthetic fractures around an anatomic cemented hip stem. Acta Orthop 94:438–446. 10.2340/17453674.2023.18263 37593786 10.2340/17453674.2023.18263 PMC10436285

[19] Girard J, Roche O, Wavreille G, Canovas F, Le Béguec P (2011) Stem subsidence after total hip revision: 183 cases at 5.9 years follow-up. Orthop Traumatology: Surg Res 97:121–126. 10.1016/j.otsr.2010.10.006 10.1016/j.otsr.2010.10.006 21435964

[20] Abdel MP, Cottino U, Larson DR, Hanssen AD, Lewallen DG, Berry DJ (2017) Modular Fluted Tapered Stems in Aseptic Revision Total Hip Arthroplasty. J Bone Joint Surg 99:873–881. 10.2106/JBJS.16.00423 28509828 10.2106/JBJS.16.00423

[21] Abdel MP, Lewallen DG, Berry DJ (2014) Periprosthetic Femur Fractures Treated With Modular Fluted, Tapered Stems. Clin Orthop Relat Res 472:599–603. 10.1007/s11999-013-2936-4 23529634 10.1007/s11999-013-2936-4 PMC3890202

[22] Perry M, Rivera J-L, Wesolowski M, Eikani C, Lack W, Cohen J et al (2023) Treatment of Vancouver B2 Femur Fractures With Open Reduction Internal Fixation Versus Revision Arthroplasty. Cureus 15:e38614. 10.7759/cureus.38614 37288216 10.7759/cureus.38614 PMC10243375

[23] Duncan CP, Masri BA (1995) Fractures of the femur after hip replacement. Instr Course Lect 44:293–304 7797866

[24] Hannon CP, Abdel MP (2023) Revision Total Hip Arthroplasty with a Modular Fluted Tapered Stem for a Periprosthetic Femoral Fracture. JBJS Essent Surg Tech 13. 10.2106/JBJS.ST.22.00023. e22.00023 10.2106/JBJS.ST.22.00023 PMC10810587 38282726

[25] Tsiridis E, Haddad FS, Gie GA (2003) The management of periprosthetic femoral fractures around hip replacements. Injury 34:95–105. 10.1016/s0020-1383(02)00257-7 12565015 10.1016/s0020-1383(02)00257-7

[26] Huang Y, Zhou Y, Shao H, Gu J, Tang H, Tang Q (2017) What Is the Difference Between Modular and Nonmodular Tapered Fluted Titanium Stems in Revision Total Hip Arthroplasty. J Arthroplast 32:3108–3113. 10.1016/j.arth.2017.05.021 10.1016/j.arth.2017.05.021 28602532

[27] Sandiford NA, Garbuz DS, Masri BA, Duncan CP (2017) Nonmodular Tapered Fluted Titanium Stems Osseointegrate Reliably at Short Term in Revision THAs. Clin Orthop Relat Res 475:186–192. 10.1007/s11999-016-5091-x 27672012 10.1007/s11999-016-5091-x PMC5174054

[28] Feng S, Zhang Y, Bao Y-H, Yang Z, Zha G-C, Chen X-Y (2020) Comparison of modular and nonmodular tapered fluted titanium stems in femoral revision hip arthroplasty: a minimum 6-year follow-up study. Sci Rep 10:13692. 10.1038/s41598-020-70626-6 32792539 10.1038/s41598-020-70626-6 PMC7426918

[29] Hannon CP, Sheehan KP, Duong SQ, Yuan BJ, Lewallen DG, Berry DJ et al (2022) Modular Fluted Tapered Stems for Periprosthetic Femoral Fractures: Excellent Results in 171 Cases. J Bone Joint Surg 104:1188–1196. 10.2106/JBJS.21.01168 35793797 10.2106/JBJS.21.01168

[30] Munro JT, Garbuz DS, Masri BA, Duncan CP (2014) Tapered Fluted Titanium Stems in the Management of Vancouver B2 and B3 Periprosthetic Femoral Fractures. Clin Orthop Relat Res 472:590–598. 10.1007/s11999-013-3087-3 23719963 10.1007/s11999-013-3087-3 PMC3890200

[31] Otero JE, Martin JR, Rowe TM, Odum SM, Mason JB (2020) Radiographic and Clinical Outcomes of Modular Tapered Fluted Stems for Femoral Revision for Paprosky III and IV Femoral Defects or Vancouver B2 and B3 Femoral Fractures. J Arthroplast 35:1069–1073. 10.1016/j.arth.2019.11.039 10.1016/j.arth.2019.11.039 31870582

[32] Lakstein D, Eliaz N, Levi O, Backstein D, Kosashvili Y, Safir O et al (2011) Fracture of Cementless Femoral Stems at the Mid-Stem Junction in Modular Revision Hip Arthroplasty Systems. J Bone Joint Surg 93:57–65. 10.2106/JBJS.I.01589 21209269 10.2106/JBJS.I.01589

[33] Passano B, Oakley CT, Lutes WB, Incavo SJ, Park KJ, Schwarzkopf R (2023) Clinical and Radiographic Outcomes of a Monoblock Fluted Titanium-Tapered Stem for Paprosky IIIa, IIIb, and IV Femoral Bone Defects. J Arthroplast 38:1342–1348. 10.1016/j.arth.2023.01.034 10.1016/j.arth.2023.01.034 36731584

[34] Gabor JA, Padilla JA, Feng JE, Schnaser E, Lutes WB, Park KJ et al (2020) Short-term outcomes with the REDAPT monolithic, tapered, fluted, grit-blasted, forged titanium revision femoral stem. Bone Joint J 102–B:191–197. 10.1302/0301-620X.102B2.BJJ-2019-0743.R1 32009430 10.1302/0301-620X.102B2.BJJ-2019-0743.R1

[35] Pomeroy E, Flynn SO, Grigoras M, Murphy TP, Stavrakis AI, Rowan FE (2022) Subsidence of monoblock and modular titanium fluted tapered stems in revision hip arthroplasty: A retrospective multicentre comparison study. J Clin Orthop Trauma 34:102021. 10.1016/j.jcot.2022.102021 36147379 10.1016/j.jcot.2022.102021 PMC9486022

[36] Pomeroy E, Lim JBT, Vasarhelyi EM, Naudie DDR, Lanting B, MacDonald SJ et al (2023) No Difference in Subsidence Between Modern Monoblock and Modular Titanium Fluted Tapered Femoral Stems. J Arthroplast 38:S223–S228. 10.1016/j.arth.2023.03.034 10.1016/j.arth.2023.03.034 36963526

[37] Rösler J, Perka C (2000) The effect of anatomical positional relationships on kinetic parameters after total hip replacement. Int Orthop 24:23–27. 10.1007/s002640050006 10774857 10.1007/s002640050006 PMC3619855

[38] Park M-S, Lim Y-J, Chung W-C, Ham D-H, Lee S-H (2009) Management of Periprosthetic Femur Fractures Treated With Distal Fixation Using a Modular Femoral Stem Using an Anterolateral Approach. J Arthroplast 24:1270–1276. 10.1016/j.arth.2009.07.013 10.1016/j.arth.2009.07.013 19729278

[39] Yu S, Saleh H, Bolz N, Buza J, Iorio R, Rathod PA et al (2020) Re-revision total hip arthroplasty: Epidemiology and factors associated with outcomes. J Clin Orthop Trauma 11:43–46. 10.1016/j.jcot.2018.08.021 32001983 10.1016/j.jcot.2018.08.021 PMC6985171

[40] Sarpong NO, Kaidi AC, Syku M, Mensah C, Blevins JL, Chalmers BP (2022) Survivorship and Risk Factors for Re-Revision after Aseptic Revision Total Hip Arthroplasty in Patients Aged ≤ 55 Years. J Arthroplasty 37:1626–1630. 10.1016/j.arth.2022.03.057 35318097 10.1016/j.arth.2022.03.057

[41] Schmalzried TP, Shepherd EF, Dorey FJ, Jackson WO, Dela Rosa M, Fa???vae F et al (2000) Wear Is a Function of Use, Not Time. Clin Orthop Relat Res 381:36–46. 10.1097/00003086-200012000-00005 10.1097/00003086-200012000-00005 11127668

